# A commentary on ‘A retrospective study: does cigarette smoking induce cervical disc degeneration?’

**DOI:** 10.1097/JS9.0000000000000602

**Published:** 2023-08-11

**Authors:** Xiang Zhang, Zhuoze Li, Hao Liu

**Affiliations:** Department of Orthopedics, Orthopedic Research Institute, West China Hospital, Sichuan University, Chengdu, People’s Republic of China

*Dear Editor*,

With great interest, we read the article entitled ‘A retrospective study: Does cigarette smoking induce cervical disc degeneration?’ written by Chen *et al*.^1^. The authors deserved to be highly praised for performing a well-designed study that included 320 participants and concluded that smoking could accelerate the process of cervical disc degeneration. This article raised some thought-provoking issues and great inspiration in clinical practice.

First of all, this article included the C1/2 cervical disc for the analysis. However, there was no C1/2 cervical disc because C1 was the atlas and C2 was the axis. Considering that this indicator (C1/2 disc segment) should not exist, we believe that addressing this issue would benefit the readers.

Second, the author provided us with clinical insights that investigating the smoking situation of patients could provide better health education for patients with neck pain and even cervical spondylosis. Considering that many patients who came to our clinic for the diagnosis and treatment of degenerative cervical diseases had a smoking history, we hope to provide more evidence to explore the relationship between smoking and intervertebral disc degeneration. We hypothesized that the presence of smoking may affect the process of cervical disc degeneration in terms of the Miyazaki disc degeneration grading system^2^. Between January 2021 and June 2021, 70 patients were randomly selected from patients who came to our hospital spine surgery outpatient clinic with a chief complaint of neck/shoulder pain. This study was approved by the Ethics Committee of Biomedical Research, West China Hospital of Sichuan University. Written informed consent was waived because of the retrospective nature of data collection (age) and the use of de-identified MRI images. The inclusion criteria and exclusion criteria were consistent with what had been described in this previous study^1^. Among all the selected patients, 25 were included in the active smoker (AS) group, 16 were included in the passive smoker (PS) group, and 29 in the negative smoker (NS) group. Among the three groups, there were no significant differences noticed in age, hypertension history, history of diabetes mellitus, body mass index (BMI), and alcoholism (*P*>0.05).

Miyazaki grades of each segment cervical spine from the three groups were recorded in Table 1. Considering the result of data analysis that the overall Miyazaki score in the NS group was lower than that of the AS and PS group, we arrived at a conclusion that was relatively close to this previous study: Smoking may accelerate the process of cervical disc degeneration. In addition, we made new discoveries as follows. The scores of C5/6 and C6/7 segments in the NS group were lower than those in the AS and PS groups (*P*<0.05), but there was no statistically significant difference in the scores of C2/3 and C3/4 segments between the NS group and the AS or PS groups, indicating that smoking may have a greater impact on C5/6 and C6/7 cervical disc degeneration. Moreover, the overall Miyazaki scores in the AS group beat those in the PS group with statistical significance (*P*=0.031), which suggested that active smoking may have a worse impact on cervical disc degeneration compared with passive smoking. Nevertheless, a larger sample size and prospective high-quality research were still needed.

**Table 1:** Comparison of Miyazaki grading of cervical disc degeneration in three groups of patients.

	C2/3					C3/4				
Group	I	II	III	IV	V	I	II	III	IV	V
AS	4	10	11	0	0	1	4	14	5	1
PS	1	4	11	0	0	0	1	11	3	1
NS	9	11	9	0	0	1	9	17	2	0
*P* value	0.138					0.338				
	C4/5					C5/6				
	I	II	III	IV	V	I	II	III	IV	V
AS	0	4	12	5	4	0	0	10	8	7
PS	0	0	11	5	0	0	0	1	13	2
NS	0	12	12	5	0	0	6	13	10	0
*P* value	0.005					<0.001				
	AS vs. PS, *P*=0.120; AS vs. NS, *P*=0.053; PS vs. NS, *P*=0.007					AS vs. PS, *P*=0.007; AS vs. NS, *P*=0.002; PS vs. NS, *P*<0.001				
	C6/7					Total Count				
	I	II	III	IV	V	I	II	III	IV	V
AS	0	0	12	8	5	5	18	59	26	17
PS	0	0	11	4	1	1	5	45	25	4
NS	1	15	9	4	0	11	53	60	21	0
*P* value	*P*<0.001					*P*<0.001				
	AS vs. PS, *P*=0.371; AS vs. NS, *P*<0.001; PS vs. NS, *P*=0.001					AS vs. PS, *P*=0.031; AS vs. NS, *P*<0.001; PS vs. NS, *P*<0.001				

*Note*: AS vs. NS, active smoker group compared to negative smoker group; AS vs. PS, active smoker group compared to passive smoker group; AS, active smoker group; NS, negative smoker group; PS vs. NS, passive smoker group compared to negative smoker group; PS, passive smoker group.

Intervertebral disc degeneration is a chronic process that is influenced by multiple factors and constantly progresses, involving multiple aspects such as gene influence, cell apoptosis, enzyme degradation, and expression of proinflammatory factors^3,4^. Therefore, we should also take a more comprehensive view of the process of intervertebral disc degeneration, and more basic studies and preclinical studies are urgently needed to further investigate the molecular mechanisms involved in the process of cervical disc degeneration and explore the effect of smoking on intervertebral disc degeneration.

In summary, our findings gave us great inspiration in clinical practice, emphasizing the importance of health education on quitting smoking for patients with neck/shoulder pain, even cervical spondylosis, which may prevent further degeneration of the intervertebral disc. In addition, as clinical doctors, we also need to pay attention to the recovery of active or passive smoking patients and prescribe personalized rehabilitation plans for them.

Once again, we highly appreciate the authors for their great effects and sincerely hope that the readers may benefit from it.

## Ethical approval

Not applicable.

## Consent

The authors provide their consent to the publication of the comments.

## Sources of funding

This comment did not receive any funding.

## Author contribution

H.L.: conceived, designed, and planned the study; X.Z. and Z.L.: wrote the manuscript.

## Conflicts of interest disclosure

The authors declare that they have no conflicts of interest.

## Research registration unique identifying number (UIN)

1. Name of the registry: not applicable.
2. Unique identifying number or registration ID: not applicable.
3. Hyperlink to your specific registration (must be publicly accessible and will be checked): not applicable.

## Guarantor

Hao Liu, e-mail: liuhao6304@126.com.
